# Comparison of saliva interleukin-2 concentration to the condition of gums in children with acute lymphoblastic leukaemia during anti-tumour treatment

**DOI:** 10.1007/s00280-015-2750-7

**Published:** 2015-05-16

**Authors:** Elżbieta Pels

**Affiliations:** Chair and Department of Paedodontics, Medical University, Lublin, Karmelicka 7 St., 20-081 Lublin, Poland

**Keywords:** Children, ALL, Gingival Index, Saliva IL-2 concentration

## Abstract

**Purpose:**

Subjects undergoing chemotherapy often develop disorders in salivation and changes in salivary composition. Therefore, a tendency to inflammatory states developing within oral mucosa is often observed. The objective of the study was to assess the effect of disturbed immunity on the gum condition in children with acute lymphoblastic leukaemia (ALL) during anti-tumour treatment and to compare saliva interleukin-2 (IL-2) concentration in relation to the condition of gums oral mucositis—must be removed in children with ALL.

**Methods:**

The study included 78 children with ALL in followed three examinations and a control group included 78 healthy children. Dental examination of the children with ALL and the control group included the evaluation of gingival condition by gingival index (GI). Children’s unstimulated saliva samples were taken, and IL-2 concentration was determined by Human IL-2 EIA.

**Results:**

Mean GI higher than 1 was observed in 3.17 % children with ALL. The results found higher frequency of gingival inflammations in children with ALL on chemotherapy compared with the healthy controls. A comparison of results for a given patient during anti-tumour therapy with regard to salivary IL-2 showed significant differences between examination 2 and examination 3. The results indicated that IL-2 level in saliva was twice higher in a patient during chemotherapy.

**Conclusions:**

An increase in salivary level of the proinflammatory IL-2 cytokine in ALL children during chemotherapy may cause pathological changes in the condition of the gums. An increase in salivary IL-2 level most probably results from a micro-injury of oral mucosa following administration of cytostatics, which in turn may cause oral *mucositis* in children with ALL.

## Introduction

Relations between immune response factors in saliva are especially important for maintenance of proper function of oral mucosa. Subjects undergoing chemotherapy often develop disorders in salivation and changes in salivary composition. Therefore, a tendency to inflammatory states developing within oral mucosa is often observed. Disorders of immunological balance lead to development of pathological lesions on the oral mucosa defined as mucositis. Mucosal barrier injury (MBI) is a frequent and difficult-to-treat complication of high-dose chemotherapy and radiotherapy, as well as haematopoietic stem cell transplantation (HSCT) [1–4].

Oxidative stress is one of potential carcinogenic mechanisms. This theory is based on studies in which free oxygen radicals may react with DNA, resulting in mutations that lead to neoplastic changes. On the other hand, reactive oxygen species are factors inducing apoptosis in mutant cells. Therefore, paradoxically, increased activity of antioxidant defence may halt elimination of mutant cells and increase promotion of a neoplastic change. Oxidative stress may also play a role in modulation of immunological reactions by activating transcription factors that control IL-2, IL-10 and TNF-α genes. Authors studying the effect of oxidative–antioxidative balance disorders in children with ALL on the level of extracellular cytokines IL-2, IL-4, IL-5, IL-10, TNF-α and INF-α in serum, observed increased IL-10 level, higher TNF-α, IL-4, IL-5 and INF-γ levels in children with ALL compared with healthy children [5].

Biological effects of IL-2 are induced by an IL-2 (IL-2R) receptor, which has three protein subunits: alpha, beta and gamma. The receptor exerts an indirect anti-tumour effect by activating NK cells, stimulating specific anti-tumour cytotoxicity and stimulating lymphokine-activated killers (LAK), which reveal non-specific and direct cytotoxic effect on cancerous cells. The basic IL-2 property is to activate effector cells to selective lysis of freshly isolated tumour cells. Apart from that, secondarily secreted cytokines, IL-1, IL-4 and IL-6, may also play a role here. The final result of this stimulation increases expression of the receptor on the membrane of helper T cells, which produce IL-2 [6–8].

## Purpose

The objective of the study was to assess the effect of disturbed immunity on the gum condition and saliva interleukin-2 (IL-2) concentration in children with acute lymphoblastic leukaemia (ALL) during anti-tumour treatment. The objective was also to compare saliva interleukin-2 concentration in relation to the Gingival Index (GI) reflecting condition of gums in children suffering from acute lymphoblastic leukaemia.

## Materials and methods

The study included 78 children aged from 2 to 18 years with acute lymphoblastic leukaemia (ALL) and a control group of 78 healthy children selected with the use of the analogous method with regard to age and sex. In the group of examined children, five had recurrent neoplasms located in the brain and spinal cord, two children had recurrent bone marrow cancer, seven children had their CNS affected, and three children had Down’s syndrome. The examination of children with ALL followed three stages: examination 1 performed prior to chemotherapy, examination 2 carried out from two days to five months following the onset of chemotherapy, examination 3 carried out after 0.5–1.5 years of anticancer therapy. Children with ALL were treated according to ALLIC BFM 2002 programme and were qualified for three risk groups: standard risk (SR), intermediate risk (IR) and high risk (HR). Verification of risk groups took place on 8, 15 and 33 days of treatment by means of bone marrow examination and assessment of leukaemia blast count: M1 < 5 %, M2 5–25 %, M3 > 25 %, assessment of response to steroid treatment and assessment of leukocytosis.

The children were treated in the Department of Pediatric Hematology and Oncology Medical University of Lublin, with strict adherence to the appropriate protocols for risk groups, in which the successive days of treatment with certain medicines are administered. The Protocol I, which was performed in all children administered: dexamethasone (DEXA), vincristine (VCR), L-asparaginase (L-ASP), daunoribicin (DNR), cyclophosphamide (CPM), cytarabine (ARA-C), 6- mercaptopurine (6-MP), 6-thioguanine (6-TG) and methotrexate (MTX). The Protocol Mm was conducted in children with SR and IR group which was administered 6-MP, MTX. Children with HR group immediately after the Protocol I had introduced HR blocks that used: DEXA, vindensin (VDS), DNR, HD MTX, ifosfamide  (IFO), L-ASP, HD ARA-C. Protocol II was then performed, which administered: VCR, doxorubicin (DOX), L-ASP, CPM, ARA-C, 6-TG, MTX or Protocol III, which administered: VCR, DOX, L-ASP, CMP, ARA-C, 6-TG, MTX. The dose was dependent on the weight, height and age of patients.

Dental examinations were performed with basic diagnostic kits in artificial light. Clinical dental examination of the children with ALL and the control group included the evaluation of gingival condition by gingival index (GI) (Löe and Silness). Two hours after morning meal, unstimulated saliva samples were taken and IL-2 concentration was determined by Human Interleukin-2 EIA DRG Diagnostics. The saliva samples were centrifuged for 15 min at 5000 rpm. Centrifuged saliva was frozen at −80 °C and stored until laboratory tests.

The results were analysed statistically. Measurable parameters were presented as means, medians, minimum, maximum and SD. *U* Man–Whitney test was used to compare two independent groups; the Spearman R correlation coefficient significance test was used to compare two quantitative traits and Wilcoxon’s test to compare dependent groups. Statistical analysis was done by STATISTICA 10.0; *p* < 0.05 was assumed statistically significant.

## Results

**Fig. 1:**
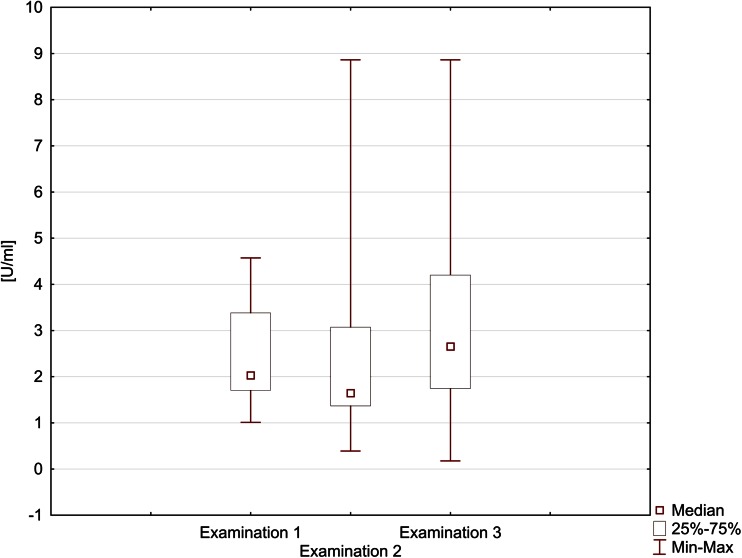
Salivary interleukin 2 (IL-2) concentration in children with ALL in subsequent examinations (U/ml)

The results of examinations in both groups and statistical analysis were listed in Table 1 and graphically presented on Fig. 1.

**Table 1:** Gingival conditions expressed as GI and saliva IL-2 concentration (in U/ml) in children with ALL and healthy controls

Studied parameter	Studied group of children		$\overline{X}$	Me	SD	*U* Man–Whitney test	
						*Z*	Significance level
GI	ALL	Examination 1	0.084	0.0	0.34	−2.5393	0.0111*
		Examination 2	0.007	0.0	0.04	−0.2360	0.8134
		Examination 3	0.017	0.0	0.13	−0.1928	0.8471
	Healthy		0.003	0.0	0.03	The above values refer to healthy children	
Saliva IL-2	ALL	Examination 1	2.38	2.0	0.90	−1.6412	0.101
		Examination 2	2.49	1.6	1.72	−0.1047	0.917
		Examination 3	3.06	2.7	1.87	−3.1538	0.002*
	Healthy		2.07	1.87	0.96	The above values refer to healthy children	

* Statistically significant value

Gingival conditions were expressed as GI. In the group of children with ALL, mean GI determined in examination 1 was 0.084 ± 0.34, in examination 2 GI = 0.007 ± 0.04 and in examination 3 mean GI = 0.017 ± 0.13. In the group of healthy controls, GI = 0.003 ± 0.03. GI values were statistically significantly higher in the group with ALL prior to treatment in comparison with the control group (Table 1). In the group with ALL, GI determined in examination 1 ranged 0–1 in 6.45 % children while GI > 1 was noted in 4.84 % children. In this group, mean GI determined over 1- to 5-month period of treatment was higher compared with the control group; however, the differences between those groups were not statistically significant. Mean GI > 1 was observed in 3.17 % children with ALL in examination 2. It can be explained by the fact that gingival inflammations occurred in that period with lesser frequency compared with examination 1. In examination 3, the children with ALL had higher GI in comparison with the control group; the differences were statistically insignificant though. Similar to examination 2, GI > 1 was noted in 3.17 % children with ALL. The results found higher frequency of gingival inflammations in children with ALL on chemotherapy compared with the healthy controls (Table 1).

Saliva concentration of interleukin-2 (IL-2) determined in the group of children with ALL in examination 1 ranged 1.01–4.57 U/ml. In that group in examination 1, mean saliva IL-2 concentration was 2.38 ± 0.90 U/ml. In the group of healthy children, saliva IL-2 concentration ranged 0.32–4.96 U/ml and mean saliva IL-2 concentration was 2.07 ± 0.96 U/ml. Mean saliva IL-2 concentrations (examination 1) were higher in the group of children with ALL in comparison with the healthy controls; however, the differences were not statistically significant (*p* > 0.05; Table 1).

In examination 2, saliva IL-2 concentrations ranged 0.39–8.86 U/ml; mean saliva IL-2 concentration was 2.49 ± 1.72 U/ml. In the group of children with ALL, mean saliva IL-2 values were higher compared with examination 1 and with the control group; however, the differences were statistically insignificant (*p* > 0.05; Table 1).

In examination 3, saliva IL-2 concentrations ranged 0.17–8.86 U/ml; mean saliva IL-2 concentration was 3.06 ± 1.87 U/ml. In the group of children with ALL, mean saliva IL-2 values were statistically significantly higher compared with the control group (*p* = 0.002; Table 1).

A comparison of results for a given patient during anti-tumour therapy with regard to salivary IL-2 showed significant differences between study 2 and study 3 (Wilcoxon signed-rank test; *Z* = 2.173; *p* = 0.0297). The results indicated that IL-2 level in saliva was twice higher in a patient during chemotherapy. The findings were also confirmed by a logistic regression model (logit): total loss: 56.4033, *χ*^2^ (3) = 9.5925 (*p* = 0.02238) (Fig. 1).

With regard to the risk group, the analysis of saliva IL-2 levels in ill children showed a statistically significant correlation between the average value for this cytokine and the risk group of children with ALL in study 1 (Spearman’s rank correlation coefficient = 0.5546, *t* = 2.4941, *p* = 0.0258) and in study 2 (Spearman’s rank correlation coefficient = 0.3988, *t* = 2.4983, *p* = 0.0176). In study 1, the lowest IL-2 values in saliva were observed in children of the SR group 2.29 ± 0.65 U/ml, and the highest average IL-2 values in saliva were observed in children of the HR group 2.44 ± 0.99 U/ml. In study 2, children from the group of SR had the highest IL-2 value and the difference between the groups was statistically significant. In study 3, children from the group of HR had again the highest IL-2 level in saliva, i.e. 3.74 ± 1.52 U/ml, although differences between the groups were close to statistical significance.

In the group of ALL children, a statistical analysis of the findings did not show significant correlations between GI and salivary IL-2 in study 1, study 2 and study 3. However, a GI increase was accompanied by an increase in saliva IL-2 in study 1 ((Spearman’s rank correlation coefficient = 0.2276, *t* = 1.4596, *p* = 0.1524) and 3 (Spearman’s rank correlation coefficient = 0.2251, *t* = 1.7445, *p* = 0.0864), which may mean that gingival inflammation is accompanied by an increase in the proinflammatory cytokine level. In study 2, a decreased GI level was accompanied by a lower level of saliva IL-2 (Spearman’s rank correlation coefficient = 0.0425 *t* = 0.2787, *p* = 0.7818).

## Discussion

In recent years, saliva has been more and more frequently used to assess biomarkers in various, sometimes serious, diseases. Comparative studies indicate that saliva may become an alternative tool to blood serum in assessment of certain biomarkers. Due to a non-invasive method of material collection, saliva is a good tool to diagnose various diseases. It enables assessment of treatment, as well as monitoring patients after treatment with little discomfort for both the patient and the physician. The use of non-invasive techniques is especially important when the patients suffer not only from the primary disease but also from its complications [9–11].

Studies confirm that certain salivary biomarkers, i.e. IFN-γ, IL-1-β1, TNF-α, IL-6, IL-4 and IL-8, may be used for predicting future development of periodontal diseases. Immunoregulatory mechanisms of cytokines involved in inflammations, infectious and immunological diseases in the oral cavity play a special role in protecting the host and maintaining oral homoeostasis. Saliva may become a good material to detect proinflammatory markers in the oral cavity [12, 13]. In the future, levels of salivary cytokines may play an important role as replacement biomarkers in assessing chemotherapy efficacy. Proinflammatory cytokines occurring in saliva may have a diagnostic and prognostic value in the future, acting as replacement markers of neoplastic transformation [14].

Research is also conducted to highlight the importance of measuring salivary cytokines to assess the risk of oral health and disease in adolescence and raise important questions about the role of salivary cytokines in intracellular and hormonal signalling. Patients undergoing chemo- and radiotherapy are especially exposed to salivation disorders. At the moment of salivary gland dysfunction, the majority of protective properties of saliva are absent [15].

Our study revealed a higher level of salivary IL-2 in children with ALL as compared to the control group and a significant increase in the average cytokine value during the anti-tumour treatment. Significant differences were observed in IL-2 levels with regard to the risk group of ALL children. In study 1, the lowest levels of saliva IL-2 were observed in children of the SR group and the highest average values of saliva IL-2 were recorded in children of HR group. Also in study 3, the children of the HR group had the highest average IL-2 value in saliva.

Du et al. [16] found that serum concentrations of H2S, IL-1β, IL-6, IL-10 and MIP-1α in children with ALL increased significantly. Meanwhile, serum concentrations of IL-2, TNF-α, IFN-γ and IL-4 decreased. After chemotherapy, concentrations of H2S and IL-10 decreased significantly, but IL-4 and IFN-γ concentrations increased markedly. At remission stage, serum concentrations of H2S, IL-1β, IL-4, IL-6, IL-10 and MIP-1α further decreased significantly, and those of IL-2, TNF-α and IFN-γ increased again. All these results suggest that H2S and cytokines are involved in immune regulatory process of children with ALL with chemotherapy, which is consistent with this report. Motowi et al. [17] suggested that measurement of serum sHLA-G might be helpful in diagnosis of ALL, while sIL-2R might be useful in diagnosis and follow-up of ALL in paediatric patients. Research carried out in order to search for new more effective treatments for patients with ALL, seems inclined to use the immune response factors, such as NK in the presence of clinical grade IL-2 and IL-15 [18].

Studies in which authors were comparing the level of proinflammatory cytokines (TNF-α, IL-1a, IL-6 and IL-8) in saliva of patients with oral squamous cell carcinoma and patients with precancerous condition of the oral cavity as well as healthy patients, revealed an increased level of these cytokines in saliva of patients with squamous cell carcinoma. The level of proinflammatory cytokines was also increased in subjects with a precancerous condition of the oral cavity in comparison with the control group [14].

Due to the general condition of the patients with ALL, there is an increased risk of inflammation in the first section of the gastrointestinal tract. Therefore, a special emphasis must be placed on prophylactics and oral hygiene. Physicians agree that the most important factor lowering the risk of oral complications is regular, at least twice-daily tooth brushing, mouth washing and effective motivation of the patient to clean dental surfaces and soft oral tissues [3, 19, 20].

## Summary

A statistically significantly higher level of gingival inflammation markers was observed in ALL children in comparison with the control group. Mean saliva IL-2 level in children with ALL was higher and statistically significantly increasing during chemotherapy in comparison with children of the control group. In the ALL group, the highest IL-2 level was observed in the HR group and the tendency was also present in the final stage of treatment.

## Conclusions

Overall, these findings suggest that the condition of gums and oral mucosa in children with ALL was not satisfactory during chemotherapy. An increase in salivary level of the proinflammatory IL-2 cytokine in ALL children during chemotherapy may cause pathological changes in the condition of the gums and potentiation of oral mucositis. An increase in salivary IL-2 level most probably results from a micro-injury of oral mucosa following administration of cytostatics, which destroy quickly dividing cells, which in turn may cause oral *mucositis* in children with ALL.
